# Annual Report of the Surgeon General U. S. A.

**Published:** 1871-12

**Authors:** 


### Annual Report of the Surgeon General. United States Army, 1871.

We copy the following statement from the Surgeon General's report, which will be instructive and interesting to our readers:

### ARMY MEDICAL MUSEUM.

The Army Medical Museum continues to increase in the number and variety of specimens and its consequent usefulness. The number of specimens added during the year was 1,516, a present total of 15,018.

The number of visitors was over 15,000 during the year.

### MEDICAL AND SURGICAL HISTORY OF THE WAR, &C.

Part First of the Medical and Surgical History of the War is near completion, and will be laid before Congress during its coming session, when it is hoped sufficient appropriation will be made to continue the publication of the remaining parts. Circular No. 4. a report upon barracks and hospitals, with a description of military posts throughout he United States, compiled by Assistant Surgeon J. S. Billings, U. S. Army; Circular No. 3, 1870, approved plans and specifications for hospital posts—also, a revised edition of the same (Circular No. 2. 1871), has been carefully revised and published during the year, and the Standard Supply Table of the Medical Department of the Army, (Circular No. 1, 1871), has been carefully revised and published with a view to more rigid responsibility and greater efficiency.

